# Effects of Komouni Formulation (Herbal Product of Persian Medicine) With a Low-Calorie Diet on Cardiometabolic Risk Factors in Overweight and Obese Women: A Triple-Blinded Randomized Clinical Trial

**DOI:** 10.5812/ijpr-136114

**Published:** 2023-06-21

**Authors:** Zahra Aghabeiglooei, Nazli Namazi, Mehrdad Karimi, Samaneh Soleymani, Mohammad Hossein Ayati, Hossein Rezaeizadeh

**Affiliations:** 1 Department of Traditional Medicine, School of Persian Medicine, Tehran University of Medical Sciences, Tehran, Iran; 2 Diabetes Research Center, Endocrinology and Metabolism Clinical Sciences Institute, Tehran University of Medical Sciences, Tehran, Iran; 3 Department of Traditional Pharmacy, School of Persian Medicine, Iran University of Medical Sciences, Tehran, Iran; 4 Endocrinology and Metabolism Research Center, Endocrinology and Metabolism Clinical Sciences Institute, Tehran University of Medical Sciences, Tehran, Iran

**Keywords:** Overweight, Obesity, Persian Medicine, Cardiovascular Diseases

## Abstract

**Background:**

The prevalence of obesity has almost tripled since 1975, and obesity places a heavy economic burden on healthcare systems. There is a high tendency to use a variety of complementary medicine modalities for weight management among obese patients. Persian Medicine is an ancient medical school practiced for thousands of years in Iran. Found in reliable Iranian traditional resources, Komouni formulation (KF) is a compound medicine that can be effective in the treatment of obesity. It comprises black caraway (*Bunium persicum* Boiss.), anise (*Pimpinella anisum* L.), fennel (*Foeniculum vulgare* Miller), and ajwain (*Trachyspemum ammi* L.)

**Objectives:**

This study aimed to determine the effects of KF on anthropometric indices and metabolic parameters in overweight and obese women.

**Methods:**

This triple-blinded randomized controlled clinical trial was performed on 70 overweight or obese women aged 20 - 40 years, with a body mass index (BMI) of 25 - 34.9 kg/m^2^. The subjects were randomly divided into two groups (each group n = 35) to receive a calorie-restricted diet with 2 g/day (500 mg 30 minutes before breakfast, 1000 mg 30 minutes before lunch, and 500 mg 30 minutes before dinner) KF or placebo for 8 weeks. Anthropometric indices, food intake, and biochemical parameters were measured at baseline and after the intervention.

**Results:**

A total of 60 women (intervention = 30; placebo = 30) completed the trial. After the intervention, the KF group experienced a significant reduction in weight (-4.8 vs. -3.2 kg; P = 0.0001), BMI (-1.8 vs. -0.79 kg/m^2^; P = 0.0001), waist circumference (-5.28 vs. -3.20 cm; P = 0.004), hip circumference (-0.018 vs. -0.008 cm; P = 0.047), fasting blood sugar (-5.6 vs. 0.33; P = 0.025), and low-density lipoprotein (-11.7 vs. 6.7; P = 0.0001), compared to the placebo group. None of the patients in the intervention and placebo groups reported any side effects.

**Conclusions:**

Using KF, along with a calorie-restricted diet, can reduce cardiometabolic risk factors in overweight and obese women. However, further studies are needed to elucidate the efficacy of KF as a complementary therapy in obesity.

## 1. Background

According to the World Health Organization, the prevalence of obesity has almost tripled since 1975, with more than 1.9 billion adults worldwide being overweight in 2016, 650 million of whom were obese (1). Obesity places a heavy economic burden on healthcare systems. It is estimated that approximately 10% of direct or indirect healthcare costs are due to obesity, and medical referral in this population is 32% more than normal-weight individuals (2). This metabolic disorder is one of the most important risk factors for several chronic diseases, including diabetes, hyperlipidemia, cardiovascular diseases, fatty liver, and some types of cancer. Moreover, it can increase susceptibility to infections, including coronavirus disease 2019 (COVID-19) (3, 4).

Treatments for obesity in conventional medicine include lifestyle modification (i.e., exercise, diet, and behavioral therapy), drug therapy, and surgery. Some anti-obesity medications have side effects, including headache, hypertension, tachycardia, insomnia, and dry mouth, especially with long-term use (5-7). Due to the lack of a definitive and completely effective treatment without side effects in conventional medicine, various complementary medicine modalities, including herbal therapy and acupuncture, are growing in popularity (8, 9).

Persian Medicine (PM) is an ancient medical school with a prolific history that has been practiced for thousands of years (10). Found in reliable PM resources, Komouni formulation (KF) is a compound medicine that can be effective in the treatment of obesity. It comprises black caraway (*Bunium persicum* Boiss.), anise (*Pimpinella anisum* L.), fennel (*Foeniculum vulgare* Miller), and ajwain (*Trachyspemum ammi* L.) (11).

According to the current evidence, the components of this product have antioxidant, hypoglycemic, anti-atherosclerosis, antidiabetic, appetite suppressant, and anti-obesity activities (12-14). No side effects have been observed following the consumption of these herbs in usual therapeutic doses (15).

## 2. Objectives

Based on previous studies, it is predicted that KF helps weight management and improvement of lipid profile. This formula has not been assessed clinically for weight management. Accordingly, this study examined the effects of KF on anthropometric indices and metabolic parameters in overweight and obese women.

## 3. Methods

### 3.1. Plant Materials

Dried anise, black caraway, ajwain, and fennel seeds were purchased from a traditional herbal shop in Tehran, Iran, and identified in the Herbarium Center of School of Pharmacy, Tehran University of Medical Sciences, under PMP-1615, PMP-1616, PMP-1617, and PMP‑1618 voucher numbers, respectively.

### 3.2. Drug Preparation

The formula and placebo capsules were prepared by Tooba Green Gold Company (Tehran, Iran). For this study, 50% hydroethanolic extracts were prepared by repeated maceration method and then freeze-dried. The volume of the resultant lyophilized powder was adjusted with pharmaceutical-grade starch (as a filler); accordingly, each 500 mg capsule contained extracts of 3 gr of fennel, 3 gr of anise, 2 gr of black caraway, and 2 gr of ajwain. The dose used in this study was consistent with the dose suggested in a valid PM textbook, *Qarabadin Salehi*. The formula mentioned in *Qarabadin Salehi* includes some components, namely *Carum carvi* (caraway), *Pimpinella anisum* (anise), *Foeniculum vulgare* (fennel), and *Trachyspermum ammi* (ajwain).

The recommended species in this formula is *Carum carvi* (caraway). Because this species grows very limitedly in Iran and is not available in the market of Iran, seeds are used in most pharmaceutical products, including the product used in this study, *Bunium persicum* (black caraway). It should be noted that in reliable PM sources, including the book *Makhzan Al-Advieh*, *Bunium persicum* (black caraway) has been introduced as an alternative to caraway due to their similar properties (11, 16).

Placebo capsules were filled with Avicel. Trace amounts of the extracts and permitted food coloring were used to match the odor and color of the formula and placebo. The capsules were placed in completely similar opaque cans. They were encoded by a coinvestigator who was not involved in the study based on the randomized allocation list.

### 3.3. Drug Standardization Based on Total Phenolic and Flavonoid Contents

Spectrophotometry was used to calculate total phenolic content. Initially, an ethanol solution of the extract (1 mg/mL) was prepared, and the reaction mixture was made by mixing ethanol solution (0.5 mL), 10% Folin-Ciocâlteu reagent (2.5 mL), and 7.5% NaHCO_3_ (2.5 mL). Subsequently, the samples were incubated at 45°C for 45 minutes. A spectrophotometer was used to determine the absorbance at 765 nm versus a blank. Gallic acid was used as a standard to construct the standard curve. Total phenolic content was expressed as mg gallic acid per milliliter of preparation (17).

Colorimetric analysis was used to calculate the total flavonoid content. The samples consisting of 1 mL ethanol solution of the extract (1 mg/mL) and 1 mL aluminum chloride 2% solution dissolved in methanol were prepared. Subsequently, the samples were incubated for 1 hour at room temperature. Absorbance was determined at 415 nm versus a blank. Standard quercetin was used to plot the calibration curve. Total flavonoid content was expressed as mg quercetin equivalent per milliliter of preparation (18).

### 3.4. Ethical Considerations

This study was approved by the Ethics Committee of Tehran University of Medical Sciences (IR.TUMS.MEDICINE.REC.1400.145) and conducted in accordance with the principles of the Declaration of Helsinki. The clinical trial protocol was registered in the Iranian Registry of Clinical Trials system (IRCT20210510051241N1).

### 3.5. Participants

This study was a triple-blinded, randomized, controlled clinical trial. Given the higher prevalence of obesity in women and their higher likelihood of using weight loss medications, this study was performed only on women (19). Recruitment posters were put up in the clinics of Tehran University of Medical Sciences and on social networks. Volunteers (n = 94) were invited to Ahmadiye Clinic (i.e., a specialized traditional medicine treatment center) for interviews from July to September 2021. A sample size of 26 per group was calculated based on a previous study due to weight changes (20), taking into account a 95% confidence interval and 95% test power. Considering a 20% probable drop-out rate, 35 participants were enrolled in each group (n = 70).

### 3.6. Random Allocation and Concealment

The participants were divided into two groups using a random allocation list generated by R4.0.2 software. To achieve concealment, the bottled KF and placebo samples were coded by a person who was not involved in the trial. The researchers, participants, and a statistician were completely blind to group allocations.

### 3.7. Inclusion and Exclusion Criteria

The inclusion criteria were women aged 20 - 40 or 40 - 50 years with normal mammography and a body mass index (BMI) of 25 - 34.9 kg/m^2^. The exclusion criteria comprised a history of cardiovascular, hepatic, renal, thyroid or pancreatic diseases, diabetes mellitus, hypertension, excessive menstrual bleeding, any type of malignancy, allergies, hormonal disorder, recent or active infectious diseases, pregnancy, lactation, or postmenopause, excessive use of decoctions and herbal medicines, smoking or alcohol consumption, a family history of breast or endometrial cancer in first-degree relatives, continuous use of acetaminophen (paracetamol), anticoagulants, or antiplatelet drugs, such as aspirin, warfarin, and heparin, and following a weight loss diet or having consumed weight loss medications in the past 6 months.

### 3.8. Study Design

All the participants read and signed a written informed consent form. At the beginning of the interview, a questionnaire for general information, including name, age, education, occupation, family history of obesity, history of previous diseases, and used medications, was completed by the researcher. Observing the principles of medical ethics and the fact that patients were not to be deprived of conventional treatments, an individualized weight loss diet was designed for all the volunteers by a nutritionist.

### 3.9. Diet Design

The amounts of dietary calories were calculated using the Mifflin equation for all the participants (21). Based on the personal characteristics and dietary habits of each participant, an individualized diet was designed. Total energy expenditure was estimated by considering the coefficient of physical activity and a 10% thermic effect of food. Since all the participants were overweight or obese, 500 kcal was deducted from the total calculated calories. The diet comprised 50%, 20%, and 30% carbohydrate, protein, and fat, respectively.

The duration of the study was 8 weeks based on similar previous studies (22, 23). Three face-to-face visits (i.e., the first visit, the end of the fourth week, and the end of the eighth week) were scheduled for the participants. At each visit, the researcher assessed anthropometric indices and food intake using a 24-hour food recall questionnaire (2 working days and 1 weekend day). Biochemical indicators and physical activity levels were also examined at the beginning of the study and the end of the trial using blood tests (after 12 hours of fasting) and the short form of the International Physical Activity Questionnaire (IPAQ), respectively.

Each participant in the intervention group received 2000 mg of KF daily (one capsule (500 mg) 30 minutes before breakfast, two capsules (1000 mg) 30 minutes before lunch, and one capsule (500 mg) 30 minutes before dinner for 8 weeks. The placebo group received the same amount of oral Avicel. The participants were instructed not to change their routine physical activity during the study and to inform the researcher of any new medications. Half of the medicines (two 60-capsule containers) were given to the participants at the beginning of the study, and the other half was delivered on the second visit. Phone calls were made once a week to reduce the risk of drop-outs, ensure the correct use of drugs, and obtain information about drug side effects. For compliance assessment, the participants were asked to deliver their capsule containers (full or empty) to the researcher on the second and third visits. The participants were excluded from the study if less than 95% of the drug was consumed.

### 3.10. Measured Variables

Anthropometric Measurements: Height and waist circumference were measured by the researcher on the first visit, and other anthropometric indices were measured on all three visits. Height was measured without shoes by a Seca stadiometer (Seca, Hamburg, Germany) with an accuracy of 0.5 cm. Weight measurement was performed by Seca scales (Seca, Hamburg, Germany) on all three visits, with an accuracy of 0.1 kg. The participants were fasting, had no shoes on, had minimum clothing, and were asked to wear the same garment in all visits.

• The BMI was calculated by dividing weight in kilograms by height in square meters.

• Waist circumference (WC) was measured with an inelastic plastic meter in the smallest circumference of the distance between the last rib and the iliac bone with an accuracy of 0.5 cm.

• Hip circumference (HC) was measured with an inelastic plastic meter in the largest HC with an accuracy of 0.5 cm.

• Wrist circumference was measured with an inelastic plastic meter exclusively from the right side at the junction of the wrist to the forearm on the styloid appendage of the ulnar bone with an accuracy of 0.5 cm.

• The waist-to-hip ratio (WHR) was obtained by dividing WC by HC.

Biochemical Evaluation: At the beginning and end of the intervention, 8 cc of blood was taken from a vein in the left arm after 12 hours of fasting and while in a sitting position. Serum was separated by centrifugation, and the mentioned factors were measured by an enzymatic method immediately after blood sampling. All the tests at the beginning and end of the study were performed using kits with the same LAT number and in the same laboratory.

Questionnaires: For physical activity measurement, the IPAQ (24) was completed by the researcher at the beginning and end of the study. The participants were accordingly divided into three groups, namely sedentary, moderately active, and highly active.

The amount of food intake was recorded during all visits by the researcher using a 24-hour food recall questionnaire (2 working days and 1 weekend day). Nutritionist IV software (version 3.5.2; First Databank Inc., Hearst Corp., San Bruno, CA, USA), modified to suit Iranian food, was used to measure the amounts of consumed macronutrients and dietary fiber.

### 3.11. Statistical Analysis

Data distribution was checked for normality using the Kolmogorov-Smirnov test. Quantitative data were expressed as mean and standard deviation. Qualitative data were presented as percentages. Intra-group comparison for the data with normal distribution was performed using the paired *t*-test. A *t*-test was used for intergroup comparison. Analysis of covariance was used to modify confounders. All the analyses were performed by SPSS software (version 26). A p-value of less than 0.05 was considered statistically significant.

## 4. Results

### 4.1. Total Phenol and Flavonoid Contents of the Formulation

**Figure 1.**
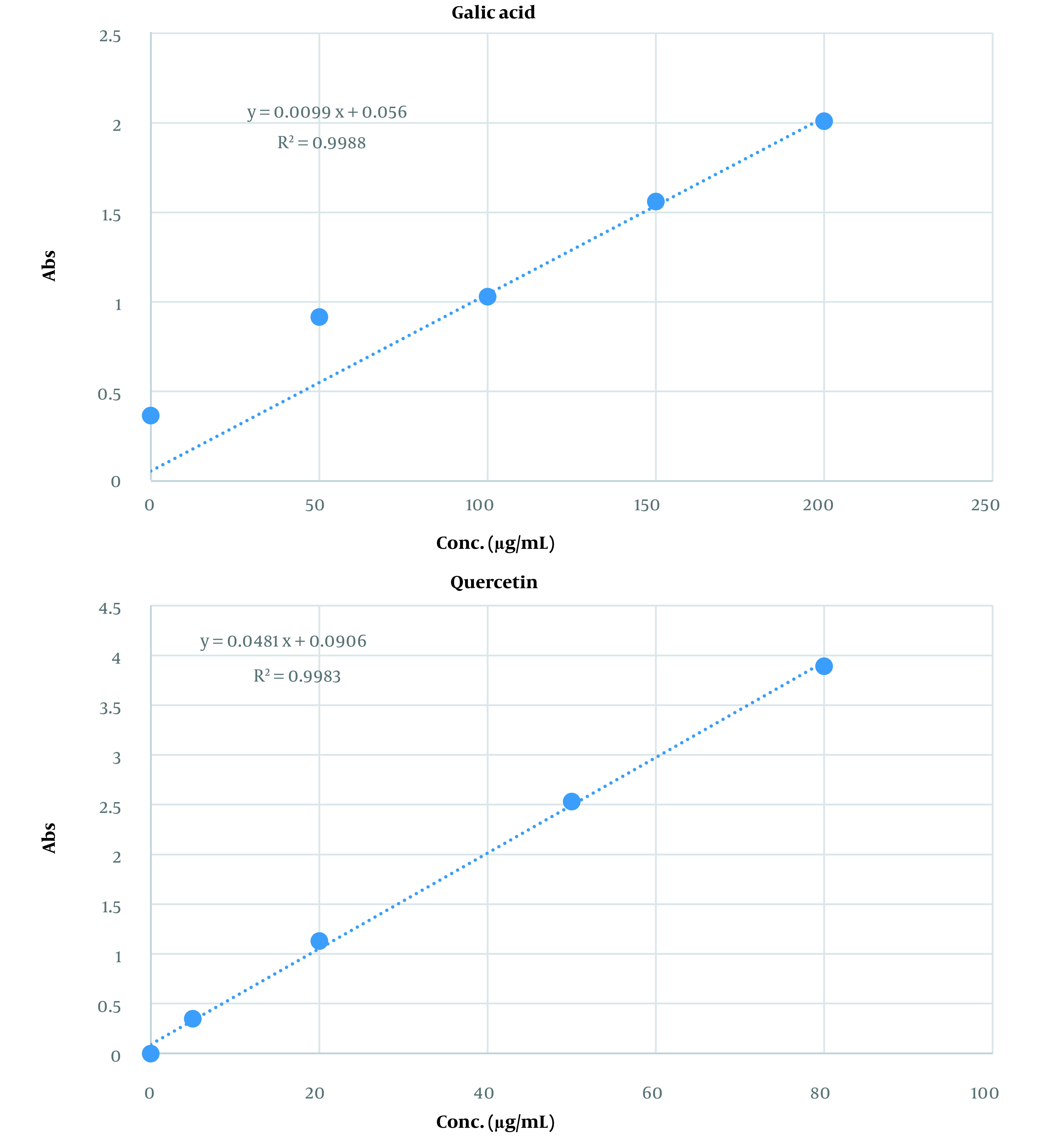
The calibration curves for gallic acid and quercetin standards

The total phenol and flavonoid contents of the extract were 96 ± 0.74 mg gallic acid equivalent/mL and 53 ± 0.48 mg quercetin equivalent/mL of extract, respectively. Figure 1 shows the calibration curves for gallic acid and quercetin standards to determine the total phenol and flavonoid contents of the capsules, respectively.

### 4.2. Demographic and Baseline Data

**Figure 2.**
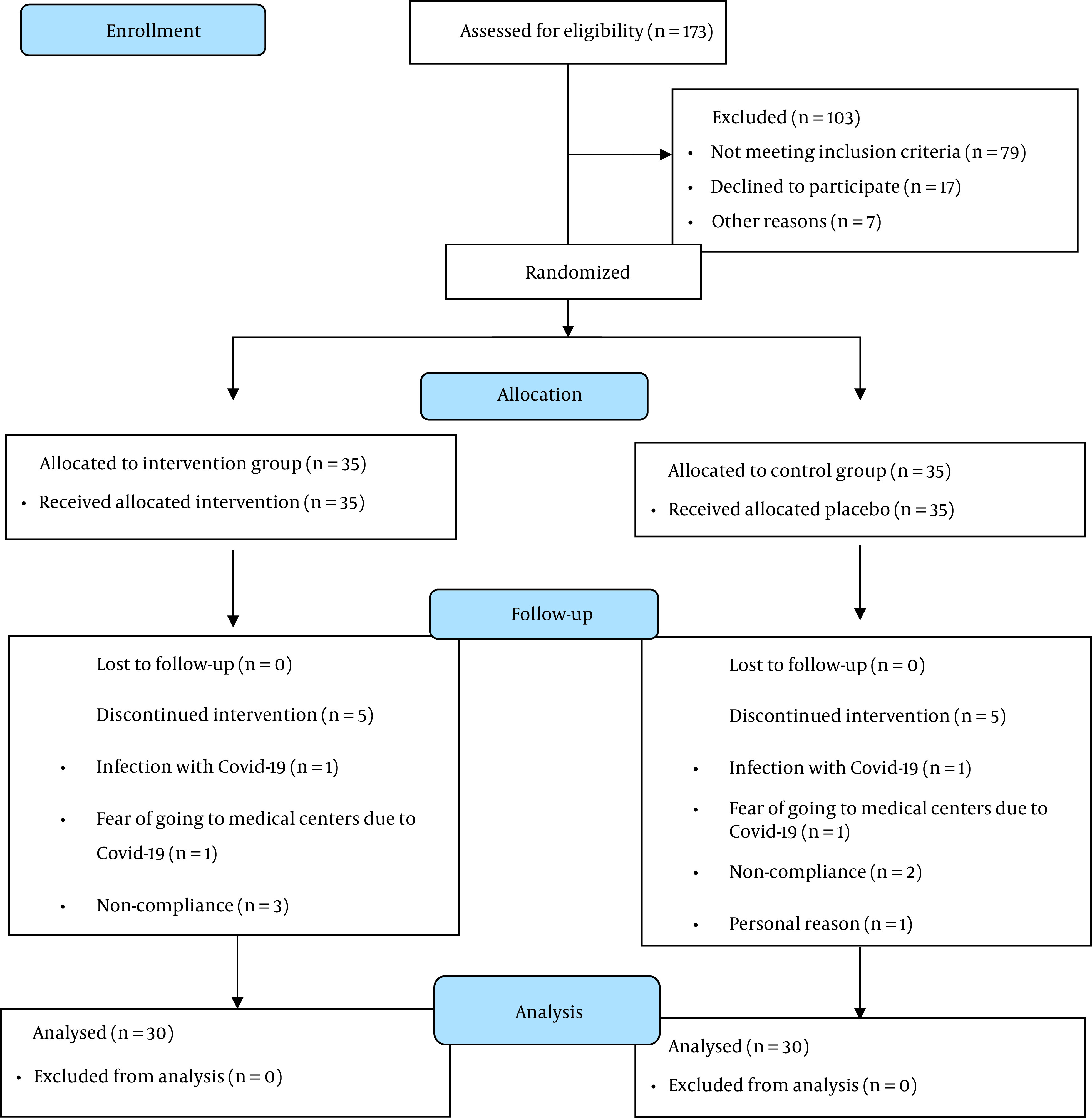
The study flowchart

A total of 70 eligible participants entered the study. Finally, 60 women (intervention = 30; placebo = 30) completed the trial (Figure 2). None of the patients in the intervention and placebo groups reported any side effects. The capsule numbers indicated high compliance of participants (> 95%).

As shown in Table 1, no significant difference was observed between the two groups regarding any demographic and baseline variables at the beginning of the study, which indicated optimal randomization. The mean values of body weight, BMI, and age of the participants were 82.4 ± 10.3 kg, 31.2 ± 2.6 kg/m^2^, and 35.2 ± 5.4 years, respectively.

**Table 1.** Baseline Characteristics of Study Groups ^a^

Variables	Komouni Group	Placebo Group	P-Value
**Age, y**	36.1 ± 5.34	34.3 ± 5.6	0.208 ^b^
**Height, cm**	161.32 ± 6.36	162.92 ± 6.13	0.325 ^b^
**Weight, kg**	81.76 ± 11.16	83.4 ± 8.47	0.536 ^b^
**Body mass index, kg/m^2^**	31.29 ± 2.56	31.38 ± 2.32	0.891 ^b^
**Dietary intake**			
Energy, kcal/d	2705.3 ± 180.1	2620 ± 207.7	0.097 ^b^
Carbohydrate, g/d	363.2 ± 73.0	359.2 ± 60.3	0.818 ^b^
Protein, g/d	105.7 ± 23.7	100.6 ± 19.0	0.364 ^b^
Total fat, g/d	94.0 ± 24.3	91.0 ± 24.4	0.626 ^b^
Fiber, g/d	20.3 ± 7.7	22.5 ± 7.2	0.265 ^b^
Iron, mg/d	27.0 ± 5.1	26.9 ± 5.7	0.965 ^b^
**Biochemical parameters**			
Cholesterol, mg/dL	197.3 ± 32.6	188.0 ± 40.5	0.715 ^b^
Triglyceride, mg/dL	137.9 ± 64.6	138.1 ± 70.0	0.934 ^b^
High-density lipoprotein, mg/dL	40.9 ± 6.5	39.3 ± 8.6	0.789 ^b^
Low-density lipoprotein, mg/dL	122.6 ± 26.1	115.9 ± 31.5	0.709 ^b^
Fasting blood sugar, mg/dL	95.8 ± 12.7	91.7 ± 5.0	0.459 ^b^
Aspartate aminotransferase, U/L	20.2 ± 9.0	20.6 ± 9.9	0.736 ^b^
Alanine transaminase, U/L	26.7 ± 20.7	26.5 ± 20.9	0.965 ^b^
**Marital status**			0.551 ^c^
Single	4 (13.3)	5 (16.7)	
Married	26 (86.7)	25 (83.3)	
**Education**			0.999 ^c^
Up to diploma	4 (13.3)	4 (13.3)	
College education	26 (86.7)	26 (86.7)	
**Family history of obesity**			0.774 ^c^
No	9 (30)	8 (26.7)	
Yes	21 (70)	22 (73.3)	
**Activity status**			0.688 ^c^
Low	27 (90)	26 (86.7)	
Moderate	3 (10)	4 (13.3)	

^a^ Values are expressed as mean ± SD or No. (%).

^b^ Independent *t*-test

^c^ Chi-square test

### 4.3. Group (Intervention Effect) Analysis

Tables 2 and 3 show changes in the studied variables in two groups of KF and placebo during the intervention. The participants did not differ significantly regarding menstrual cycle at different measurement times. As shown in Table 2, there was a significant decrease in the body composition indices, including weight, BMI, WC, and HC, in both groups at the end of the intervention; however, the KF group had a more substantial reduction than the placebo group in both weight (-4.8 vs. -3.2 kg; P = 0.0001) and BMI (-1.8 vs. -0.79 kg/m^2^; P = 0.0001). In both indices, WC and HC, the KF group showed more changes than the placebo group (WC: -5.28 vs. -3.20 cm; P = 0.004) (HC: -0.018 vs. -0.008 cm; P = 0.047).

**Table 2.** Comparison of Anthropometric Indices at Baseline and After the Intervention

Variables	Komouni Group			Placebo Group			P-value	
	Before	After	P-value ^a^	Before	After	P-value ^a^	Independent *t*-test	ANCOVA ^b^
**Body composition**								
Weight, kg	81.76 ± 11.16	76.97 ± 10.68	0.0001 ^c^	83.4 ± 8.47	81.33 ± 8.73	0.0001 ^c^	0.0001 ^c^	0.001 ^c^
Body mass index, kg/m^2^	31.29 ± 2.66	29.46 ± 2.62	0.0001 ^c^	31.38 ± 2.33	30.59 ± 2.3	0.0001 ^c^	0.0001 ^c^	0.0001 ^c^
**Anthropometric indices**								
Waist circumference, cm	91.93 ± 8.36	86.65 ± 7.87	0.0001 ^c^	92.52 ± 7.49	89.32 ± 7.63	0.0001 ^c^	0.004 ^c^	0.004 ^c^
Hip circumference, cm	111.4 ± 6.6	107.3 ± 6.6	0.0001 ^c^	114.4 ± 6.1	111.6 ± 6.8	0.0001 ^c^	0.047 ^c^	
Waist-to-hip ratio	0.83 ± 0.06	0.81 ± 0.06	0.0001 ^c^	0.81 ± 0.05	0.8 ± 0.05	0.013 ^c^	0.07	0.130

^a^ Paired *t*-test.

^b^ Analysis of covariance (adjusted for high-density lipoprotein, low-density lipoprotein, cholesterol, energy, dietary intake changes, and baseline values).

^c^ Significant at 0.05.

**Table 3.** Comparison of Biochemical Parameters and Dietary Intake at Baseline and After the Intervention

Variables	Komouni Group			Placebo Group			P-Value
	Before	After	P-Value	Before	After	P-Value	
**Lipid and atherogenic profile**							
Cholesterol, mg/dL	379.63 ± 358.73	232.01 ± 218.28	0.0001 ^a, b^	332.53 ± 354.69	237.59 ± 240.87	0.0001 ^a, b^	0.652 ^c^
Triglyceride, mg/dL	137.93 ± 64.63	140.77 ± 67.96	0.707 ^b^	138.13 ± 70.05	143.23 ± 58.89	0.0001 ^a, b^	0.84 ^c^
HDL, mg/dL	40.9 ± 6.5	37.93 ± 6.48	0.011 ^a, b^	39.3 ± 8.62	38.53 ± 8.85	0.0001 ^a, b^	0.123 ^c^
LDL, mg/dL	3.09 ± 0.89	3 ± 0.71	0.003 ^a, b^	3.09 ± 1.05	3.35 ± 1.09	0.0001 ^a, b^	0.0001 ^a, c^
LDL-to-HDL ratio	3.09 ± 0.89	3 ± 0.71	0.355 ^b^	3.09 ± 1.05	3.35 ± 1.09	0.003 ^a, b^	0.007 ^a, c^
Cholesterol-to-HDL ratio	4.95 ± 1.14	4.75 ± 0.92	0.144 ^b^	4.98 ± 1.4	5.2 ± 1.44	0.072 ^b^	0.022 ^a, c^
Fasting blood sugar, mg/dL	95.8 ± 12.7	90.2 ± 8.4	0.009 ^a, b^	91.7 ± 5.0	91.4 ± 7.7	0.770 ^a, b^	0.026 ^a, c^
Aspartate aminotransferase, U/L	20.2 ± 9.0	17.6 ± 5.6	0.142 ^a, b^	20.6 ± 9.9	18.4 ± 8.5	0.297 ^a, b^	0.861 ^c^
Alanine transaminase, U/L	26.7 ± 20.7	26.3 ± 10.9	0.896 ^a, b^	26.5 ± 20.9	26.8 ± 11.1	0.954 ^a, b^	0.896 ^c^
**Energy and macronutrients consumption**							
Energy, kcal/d	2705.3 ± 180.17	1770.1 ± 212.9	0.0001 ^a, b^	2620.6 ± 207.79	1908.27 ± 131.02	0.542 ^b^	0.0001 ^a, c^
Carbohydrates, g/d	363.22 ± 73.09	221.6 ± 52.82	0.0001 ^a, b^	359.23 ± 60.31	249.86 ± 61.63	0.928 ^b^	0.723 ^c^
Protein, g/d	105.76 ± 23.77	71.22 ± 16.4	0.0001 ^a, b^	100.67 ± 19.05	82.6 ± 16.59	0.666 ^b^	0.011 ^a, c^
Total fat, g/d	94.1 ± 24.3	59.54 ± 17.54	0.0001 ^a, b^	91.01 ± 24.49	61.75 ± 22.1	0.592 ^b^	0.529 ^c^
**Micronutrients consumption**							
Fiber	20.37 ± 7.78	15.24 ± 5.9	0.009 ^a, b^	22.55 ± 7.24	16.52 ± 5.58	0.332 ^b^	0.725 ^c^
Iron	27.01 ± 5.16	18.21 ± 4.08	0.0001 ^a, b^	26.95 ± 5.73	20.51 ± 4.53	0.415 ^b^	0.145 ^c^
Calcium	932.25 ± 225.16	763 ± 330.42	0.038 ^a, b^	900.51 ± 200.8	802.52 ± 294.31	0.337 ^b^	0.50 ^c^
Cobalamin	20.37 ± 38.39	16.56 ± 33.31	0.668 ^b^	15.58 ± 39.48	17.59 ± 33.18	0.450 ^b^	0.664 ^c^
Vitamin A	4257.86 ± 4893.51	3556.07 ± 2748.49	0.472 ^b^	4325.68 ± 4917.56	3560.99 ± 2771.44	0.977 ^b^	0.965 ^c^
Vitamin D	0.86 ± 0.78	0.58 ± 0.69	0.114 ^b^	1.02 ± 0.7	0.71 ± 0.89	0.982 ^b^	0.944 ^c^

Abbreviations: HDL, high-density lipoprotein; LDL, low-density lipoprotein.

^a^ Significant at 0.05

^b^ Paired *t*-test

^c^ Independent *t*-test

The serum levels of triglyceride (2.83 vs. 5.10; P = 0.84) and high-density lipoprotein (HDL) (-2.97 vs. -0.76; P = 0.123) decreased significantly in both groups, compared to the baseline (P < 0.05); nevertheless, the changes were not significant between the two groups. The two groups differed significantly in terms of fasting blood sugar (FBS) (-5.6 vs. 0.33; P = 0.025), low-density lipoprotein (LDL) (-11.7 vs. 6.7; P = 0.0001), LDL-to-HDL ratio (-0.09 vs. 0.26; P = 0.007), and cholesterol-to-HDL ratio (-0.20 vs. 0.22; P = 0.022) at the end of the intervention.

After the adjustment of energy, dietary intake, baseline values, cholesterol, HDL, and LDL, some variables, including weight, WC, HC, and BMI, reduced significantly in the KF group compared to the placebo group after the intervention (P < 0.05) (Table 2). Liver enzymes did not significantly change with the intervention (Table 3).

Since both groups adhered to the diet throughout the trial, energy intake decreased significantly in both groups (P < 0.05) (Table 3). Furthermore, the overall effect of the intervention on calories (P = 0.0004) was statistically significant. Accordingly, in the eighth week of the study, the KF group received an average of 195 kcal less than the placebo group (Table 3).

The recommended and consumed calories and macronutrients were compared to evaluate adherence to the diet. As shown in Table 4, there was a slight difference between the recommended and consumed intake.

**Table 4.** Comparison of the Recommended and Consumed Calories and Macronutrients to Evaluate Adherence to Diet ^a^

Variables	4th Week		8th Week	
	Komouni Group	Placebo Group	Komouni Group	Placebo Group
**Energy, kcal/day**				
Recommended	1779.1 ± 219.9a	1830.3 ± 179.3	1779.1 ± 219.9	1830.3 ± 179.3
Consumed	1742 ± 209.4	1908.2 ± 131.0	1698.6 ± 217.5	1866.4 ± 163.1
P-value ^b^	0.458	0.057	0.183	0.435
**Protein, % of total energy**				
Recommended	20 ± 0	20 ± 0	20 ± 0	20 ± 0
Consumed	19.0 ± 7.5	16.0 ± 2.5	16.8 ± 4.0	18.5 ± 6.1
P-value	0.506	0.000	0.000	0.204
**Carbohydrate, % of total energy**				
Recommended	50 ± 0	50 ± 0	50 ± 0	50 ± 0
Consumed	50.9 ± 10.8	53.5 ± 10.7	51.6 ± 8.7	52.3 ± 11.2
P-value	0.628	0.079	0.307	0.259
**Total fat, % of total energy**				
Recommended	30 ± 0	30 ± 0	30 ± 0	30 ± 0
Consumed	30.1 ± 10.2	30.1 ± 9.7	31.3 ± 8.3	29.3 ± 10.4
P-value	0.930	0.926	0.391	0.716

^a^ Values are expressed as mean ± SD.

^b^ Paired *t*-test, normality checked

## 5. Discussion

This triple-blinded randomized clinical trial evaluated the effects of KF on cardiometabolic factors of overweight and obese women. The results indicated that daily consumption of 2000 mg of KF, along with a calorie-restricted diet, can significantly reduce weight, WC, HC, FBS, and LDL. To the best of our knowledge, no study has investigated the effects of this product on cardiometabolic factors. However, there is evidence for the beneficial effects of each component with controversial results on blood glucose, lipid profile, and weight loss in animal and human studies.

In a clinical trial in 2013, Kazemipoor et al. investigated the anti-obesity effects of caraway in obese women. At the end of the study, the intervention group showed a significant decrease in weight, BMI, fat percentage, and WHR. However, the lipid profile did not alter significantly (25). Significant weight loss following the consumption of caraway extract was in line with the results of the present study; however, the insignificant effect on lipid profile was contrary to the current study’s findings. There are two possible explanations for this inconsistency. Firstly, unlike the present study, a calorie-restricted diet was not prescribed in Kazemipoor et al.’s study. Secondly, the three other herbal components in KF contribute to improvement in lipid profiles and formula effectiveness.

A clinical study revealed that aromatherapy with fennel reduces appetite in obese individuals (26). Based on a single-blinded randomized controlled trial, daily consumption of 10 gr of ajwain decreases LDL and increases HDL (27). The reason for the controversy in the results of these studies might be due to the differences in the health status of the studied population and underlying diseases, gender, drug dosage, intervention duration, method of administration, level of physical activity, and diet. Additionally, including four medicinal plants in KF might increase drug effectiveness due to synergistic effects.

Based on the results of numerous articles, black caraway has antioxidant, hypoglycemic, weight-reducing, antihypertensive, and antidyslipidemic properties. Possible mechanisms include (1) the reduction of ghrelin (appetite-stimulating hormone); (2) the reduction of production of visceral fat and, thereby lowering pro-inflammatory cytokines produced in these tissues (by carvacrol) (28-31).

Anise has been shown to have antioxidant, antidiabetic, anti-inflammatory, anti-atherosclerotic, anti-obesity, antihypertensive, and lipid-lowering effects. One of the possible mechanisms of these properties is the reduction of triglyceride, saturated fats, and cholesterol (12, 13, 32). Trans-anethole, a substance extracted from fennel’s essential oil, is structurally similar to catecholamines and acts similarly to amphetamines in controlling appetite. It is one of the possible mechanisms that fennel causes weight loss (14, 22, 26). The lipid-lowering effect of ajwain is proposed to be due to improved lipoprotein catabolism, inhibition of HMG-CoA reductase, and inhibition of lysosomal lipid hydrolytic enzymes secreted by the liver (33-35).

Based on previous studies on the four above-mentioned medicinal plants and possible mechanisms, it is predicted that this formula might help weight management in obese and overweight individuals via the synergistic effects of mechanisms. These effects include appetite suppression via the reduction of ghrelin, increase in conjugated linoleic acid (both suppressing appetite and lowering blood lipids) and trans-Anethole, improvement in lipoprotein catabolism, inhibition of HMG-CoA reductase, and inhibition of lysosomal lipid hydrolytic enzymes. Additionally, the herbs used in this formula have a hot-dry temperament (Mizaj) based on PM. The possible PM-based mechanism includes the drying action of the comprising herbs, which dissolves excessive moisture and phlegmatic substances in the gastrointestinal tract and other body parts, thereby reducing body fat. Via a reinforcing action on digestion and the gastrointestinal system, they also improve body metabolism and reduce the production of phlegmatic substances and fat. This formula may eliminate two of the most important causes of obesity, namely low metabolism and poor digestion (36).

### 5.1. Study Limitations

The most important limitation of this study was the difference in the lifestyle of the participants. This study evaluated these differences to some extent by using a physical activity questionnaire and a food diary. However, according to PM, consuming similar calories is not necessarily enough because each food’s hot or cold nature can induce different effects in individuals (36). One of the most important current restrictions was the COVID-19 pandemic and challenges in transportation, especially to the medical center and laboratory. Therefore, to prevent unnecessary trips, the blood samples were taken by a laboratory representative at the clinic.

### 5.2. Conclusions

Using KF, along with a calorie-restricted diet, for 8 weeks can reduce cardiometabolic risk factors in overweight and obese women. It is recommended that future studies investigate the effects of KF on metabolic factors at the cellular and molecular levels, intestinal microbiomes, hormonal status, and oxidative stress in obese patients.

## Data Availability

The dataset presented in the study is available on request from the corresponding author during submission or after publication. The data are not publicly available due to ethics.

## Clinical Trial Registration Code:

The clinical trial protocol was registered in the Iranian Registry of Clinical Trials system (IRCT20210510051241N1).
